# Does the Excellent Enactment of Highest Strengths Reveal Virtues?

**DOI:** 10.3389/fpsyg.2020.01545

**Published:** 2020-07-22

**Authors:** Fiorina Giuliani, Willibald Ruch, Fabian Gander

**Affiliations:** Personality and Assessment, Department of Psychology, University of Zurich, Zurich, Switzerland

**Keywords:** character strengths, virtues, VIA classification, fulfillment, intellectual quality, moral quality, immorality

## Abstract

Two studies examined the assumption that character strengths enable virtues and facilitate the good life. Study 1 validated a “layperson’s excellent enactment of highest strengths paradigm”. This paradigm states that more appropriate assignments of character strengths to virtues are obtained when based on descriptions of highest character strengths enacted in an excellent way, than when based on lowest character strengths, or typical enactments. A sample of *N* = 230 German-speaking participants provided descriptions of situations in which they enacted their highest and lowest strengths excellently and typically and rated these situations on the degree of the six core virtues, strength expression, fulfillment, and intellectual and moral quality. Behavior examples of highest strengths excellently enacted were rated higher and with higher differentiation in the dependent variables than typical enactments or lowest strengths, thus confirming the paradigm. In Study 2, we applied the paradigm: A second sample of *N* = 113 German-speaking participants rated a selected subset of strengths–behaviors of layperson’s excellent enactment of highest strengths collected in Study 1 in regard to their degree of the six core virtues. Results confirmed previous convergent and discrepant findings with the theoretical VIA classification. We can conclude that the excellent enactment of highest strengths does indeed reveal virtues. Future studies should use the paradigm and examine culturally diverse samples with different methods for further examining the VIA classification.

## Introduction

In 2004, Peterson and Seligman introduced the VIA classification: a hierarchical classification of 6 virtues and 24 corresponding character strengths. Modeled on the Linnaean classification of species, the VIA classification (see Supplementary Appendix A) is composed of three conceptual levels ranging from the abstract to the specific: (1) virtues, which are defined as core characteristics valued by moral philosophers and religious thinkers, are the most abstract entries of the classification; (2) character strengths, morally valued traits that define the virtues; and (3) situational themes, specific habits that allow people to manifest given character strengths in present situations.

For the development of the VIA classification, researchers followed three steps. First, they searched for culturally and historically ubiquitous virtues and found six universal virtues (Dahlsgaard et al., 2005), which are wisdom and knowledge, courage, humanity, justice, temperance, and transcendence. Second, they generated and defined character strengths by applying up to 12 criteria (Peterson and Seligman, 2004; Ruch and Stahlmann, 2019): the trait must (1) be ubiquitous, (2) contribute to various fulfillments, (3) be morally valued in its own right, (4) not diminish other people, (5) have a non-felicitous opposite, (6) be trait-like in that it is stable over time and across situations, (7) measurable, (8) be distinct from other positive traits, (9) be embodied in consensual paragons, (10) have observable prodigies, (11) be possibly non-existent in some people, and (12) be sustained in the larger society by institutions and rituals intended to cultivate it. Third, they assigned the character strengths to the corresponding virtues based on theoretical considerations. They argued that character strengths are “the psychological ingredients—processes or mechanisms—that define the virtues” (p. 13). In other words, character strengths are “distinguishable routes to displaying one or another of the virtues” (p. 13). For example, the virtue of wisdom and knowledge can be achieved through creativity, curiosity, love of learning, open-mindedness, and perspective. Character strengths of a virtue also share a common function: wisdom and knowledge, for example, is composed of “cognitive strengths that entail the acquisition and use of knowledge” (pp. 29–30).

So far, only a few studies have empirically examined the assignment of character strengths to virtues, even though the study of this link is highly important, for three main reasons. First, the assignment of character strengths to virtues is the theoretical core assumption in the VIA classification. Second, as Peterson and Seligman (2004) suggested, virtues can be displayed and achieved by the application of various character strengths, and it is our goal to empirically detect these character strengths. Third, from a practical point of view, the knowledge of the classification forms the basis for the development of programs aimed at cultivating good character. More precisely, people can be encouraged to practice applying character strengths (a character strength of each virtue) in an excellent way, which in turn should lead to a reinforcement of the six virtues, and consequently results in the development of good character. With the present set of studies, we aim to provide more empirical information about these important theoretical assumptions. We aim to empirically study this assignment, based on the enactment of character strengths in specific situations, that is, character strengths–behaviors. Before that, however, we need to determine how characteristics of character strengths and virtues can be best investigated. Specifically, for our study aim, we need to identify the best suited (most appropriate) strengths–behaviors for the study of the assignment of strengths to virtues that should yield the most valid results. For that, we are going to establish a “layperson’s excellent enactment of highest strengths paradigm.”

### Previous Studies Testing the Link Between Character Strengths and Virtues

Two previous publications tested the proposed classification of character strengths to virtues. In the first publication (Ruch and Proyer, 2015), 70 experts from psychology, philosophy, and theology, and 41 laypeople rated how prototypical the strengths are for each of the six virtues. The results supported the validity of the classification, with participants indicating that the strengths were very good (open-mindedness, love of learning, perspective, bravery, love, kindness, fairness, self-regulation, and spirituality), good (creativity, curiosity, persistence, honesty, zest, social intelligence, teamwork, modesty, prudence, beauty, and hope), or acceptable markers (leadership, forgiveness, and gratitude) of their virtues. Only one strength, humor, failed to reach the cutoff score for its assigned virtue (transcendence). Humor seemed to be a marker for humanity, but it was also prototypical for wisdom. A few other strengths were also found to be stronger indicators of different virtues than the one they were initially assigned to: Teamwork and gratitude were more prototypical for humanity; forgiveness was more prototypical for humanity and justice; and leadership was more relevant for courage and for wisdom. Furthermore, four character strengths marked their own virtue best, but were also good markers for another virtue. This was the case for honesty, which also marked justice; social intelligence and prudence, which also marked wisdom; and fairness, which also marked humanity.

In the second publication on this topic, Ruch et al. (2019) tested the connection between character strengths and virtues in two studies: In the first study, German-speaking laypeople wrote short behavioral descriptions of both a typical and an excellent example of their highest character strength (determined by the highest-ranking strengths based on the results of a self-report questionnaire, the VIA-IS; Ruch et al., 2010). For excellent enactments, Ruch et al. (2019) instructed participants to write about enactments in which they were able to bring the strength to “fully bloom”, to show it in a particularly outstanding way, and to use it to a very high degree. In contrast, for typical enactments, participants were asked to write about enactments in which they showed the strength to a lesser extent and used it in the typical way just like in everyday situations. Participants were then asked to score the strengths–behaviors in terms of their degree of virtuousness (i.e., the degree of wisdom, courage, humanity, justice, temperance, and transcendence). The second study (Ruch et al., 2019, study 2) tested the common features (functions) of character strengths. Though all strengths corresponding with a given virtue are distinct, they are expected to serve a common function, i.e., strengths of wisdom and knowledge should serve the same purpose, namely, the acquisition and use of knowledge. German-speaking participants indicated to what degree each of the 24 character strengths fulfilled its purported function. For each character strength, the rating on the originally assigned virtue was compared with the average ratings of the other virtues in both studies.

Overall, the results of Ruch et al. (2019) corresponded fairly with the VIA classification (correlations of mean ratings: *r* = 0.38 and *r* = 0.50 for study 1 and study 2, respectively) and Ruch and Proyer (2015); *r* = 0.68 and *r* = 0.72) results. The findings of both studies of Ruch et al. (2019) and the study by Ruch and Proyer (2015) were in line with each other for 16 out of the 24 character strengths; these 16 strengths received the highest rating for the same virtue across all three studies, while for 13 of these 16 character strengths, the virtue corresponded with the original assignment of Peterson and Seligman (2004). The exceptions were the character strengths of forgiveness, gratitude, and humor that received the highest ratings for the virtue of humanity, in disagreement with the assignment by Peterson and Seligman (2004). If these results are replicated using different methods and culturally more diverse samples, then one could start a discussion of a potential reclassification of strengths and address these strengths first. For the remaining eight character strengths, the assignment to virtues corresponded in two out of the three studies; for six of these strengths, the assignment was in line with the VIA classification. Thus, for two further character strengths— teamwork and leadership—a reclassification (to the virtues of humanity and courage, respectively) might be considered.

### The Layperson’s Excellent Enactment of Highest Strengths Paradigm

Overall, these studies advanced the empirical validation of the VIA classification considerably. The links between strengths and virtues should generalize across different methods of assessment, but only a few methods of assessment were utilized so far. While there is some convergence, we nonetheless argue that the previous studies were limited in some regards: Two of the three studies (i.e., Ruch and Proyer, 2015; Ruch et al., 2019, study 2) used a very abstract approach to verify the VIA classification by analyzing the correspondence of virtues and character strengths at the level of abstract concepts. That is, participants were directly asked whether a strength is a good example of a virtue, or whether a strength fulfills a function associated with a virtue. This seems a rather difficult task also for expert raters, and assignments could be influenced by lay conceptualizations of the virtues or by lay conceptualizations of the associations between strengths and virtues. We argue that specific strengths–behaviors as employed by Ruch et al. (2019), study 1 should be considered instead of abstract concepts. In their study, the same participants provided the strengths–behaviors and the ratings of the six core virtues. However, for obtaining a more objective assessment it would be better if one group of people provides the strengths-behaviors while another group provides the virtue ratings.

Furthermore, when analyzing the assignment of character strengths to virtues on the level of specific behaviors, one could hypothesize (in line with Ruch et al., 2019) that the most valid results should be obtained when focusing on specific behavioral examples in which a strength was shown to a very high degree. When reflecting upon which people would be best to provide such behavior examples, one might consider those people who possess a strength to a very high degree: They should have a profound knowledge of this strength and have a rich history of displaying this strength, also to a high degree, and/or in an excellent manner.

Therefore, we argue that the next step in the empirical evaluation of the VIA classification should be based on strengths–behaviors that are rated in terms of their virtuousness (with regard to the core virtues) by people unrelated to those who provided the strengths–behaviors for obtaining a more objective and scientifically more rigorous picture. However, the expectation that strength enactments of high scorers in an excellent way are more valid for the study of the assignment of strengths to virtues should be empirically examined. Thus, we propose a “layperson’s excellent enactment of highest strengths paradigm”, which assumes that the most appropriate assignments of strengths to virtues are obtained when strengths–behaviors are provided by individuals who possess the strength of interest to a high degree or when individuals display the strength in an excellent manner.

This paper presents two studies, which aim at expanding upon the studies by Ruch and Proyer (2015) and Ruch et al. (2019). In study 1, we aim to evaluate the assumptions underlying the “layperson’s excellent enactment of highest strengths paradigm” and demonstrate that enactments of a strength that are best suited for the study of the assignment of strengths to virtues can be found for individuals’ highest strength compared to individuals’ lowest strength. Additionally, excellent enactments of strengths should be more appropriate compared to typical enactments of strengths. After having evaluated this paradigm, it will be possible to select strengths–behavior examples that are best suited for studying the association of character strengths to virtues in study 2. While in study 1, the raters judge the degree of the six virtues of their self-experienced enactments of strengths, the raters in study 2 are unaffiliated with the persons who provided the strengths–behaviors examples, and will judge the degree of the virtues based solely on the written material. Thus, the most appropriate examples of strengths–behaviors identified in study 1 are used to verify the VIA classification.

The present set of studies differs from earlier studies (Ruch and Proyer, 2015; Ruch et al., 2019) in two main regards: First, we examine what kind of informants (i.e., high vs. low scorers) and what type of information (i.e., excellent vs. typical enactment) yield the most appropriate information about strengths (study 1). Second, we collected core virtue ratings of people unaffiliated with those who provided the strengths–behaviors descriptions (study 2).

## Study 1

Study 1 aims at validating the “layperson’s excellent enactment of highest strengths paradigm”. We suggest that if the properties of character strengths are examined on the basis of strengths–behaviors, one should only examine (i) persons who “possess” the strength of interest to a high degree, and (ii) behavior examples, in which these strengths were shown to a very high (i.e., excellent) degree. For the purpose of testing this assumption, we compared behavior examples of excellent and typical enactments of people who do have a strength to a very high degree (i.e., the strength is their individual highest-ranking strength) and people who do have a strength to a very low degree (i.e., the strength is their individual lowest ranking strength).

We set up six criteria [(1) Degree of strengths expression, (2) Fulfillment, (3) Morality, (4) Virtuousness, (5) Differentiation between core virtues, (6) Consistency] to answer the question whether ratings of excellent strengths–behaviors provided by high scorers are more appropriate for studying the assignment of strengths to virtues:

First, the manifestation of character strengths should be higher in more appropriate strengths–behaviors, that is, the strength of interest should be displayed clearly in the behavioral act; otherwise, situational influences might bias the ratings of virtuousness. Therefore, we asked participants to rate the degree to which a strength was shown in the described strengths–behaviors (“degree of strengths expression”).

Second, since strengths should “contribute to various fulfillments which constitute the good life, for oneself and for others” (Peterson and Seligman, 2004; p. 17), we expect excellent strengths–behaviors of high scorers to be most fulfilling. Various studies have confirmed the robust relationships between character strengths and different aspects of the good life (e.g., Park et al., 2004; Hausler et al., 2017; Wagner et al., 2020), as consequences of these fulfillments. In the present study, participants were asked to indicate the degree of fulfillment they experienced while enacting the strength. By definition, strengths–behaviors best suited for the study of the assignment of character strengths to virtues should be rated as more *fulfilling* than strengths–behaviors that are less suited for the study of the assignment of character strengths to virtues.

Third, “each strength is morally valued in its own right, even in the absence of obvious beneficial outcomes” (Peterson and Seligman, 2004; p. 18) and therefore appropriate strengths–behaviors should be rated high in morality. As Peterson and Seligman’s definition of morality follows Aristotle’s ideas on morality, we decided to use Aristotle’s concept of morality, which distinguishes between the two components “intellectual and moral quality.” We asked participants about the moral quality of the strengths–behaviors, in line with Aristotle (2000) ideas: Moral quality refers to the heart and is characterized by a high degree of selflessness, charity, and self-control. It helps to act morally and ethically. A person who acts with moral quality is responsible and has the well-being of others in mind. Since Aristotle (2000) distinguished between moral and intellectual excellence, we also asked about the intellectual quality of the strengths–behaviors. Intellectual quality refers to the intellect and is characterized by a high degree of knowledge, paired with intellect and life experience. It helps to properly assess specific situations and to find suitable ways and means to do the right thing. Furthermore, we also asked participants whether the strengths–behaviors were free of immorality, to ensure that enactments do not include immoral elements, but would rather be fully morally valued. Immorality can be described as something reprehensible, as a shameful act, or as a bad habit. These behaviors cause harm to individuals, groups, and societies. Immorality does not refer to pathological behaviors, but to immoral and unethical ones (see Beermann and Ruch, 2009).

Fourth, since strengths theoretically represent different ways of displaying the core virtues, more appropriate strengths–behaviors should be considered more virtuous by showing a higher degree of wisdom and knowledge, courage, humanity, temperance, and transcendence.

Fifth, more appropriate strengths–behaviors should make it possible to distinguish more clearly between the patterns of core virtue ratings. Therefore, we have also analyzed the degree of *differentiation* among the core virtue ratings. Similar ideas have been brought forward with regard to vocational interests—for example, having more strongly differentiated profiles of vocational interests goes along with more stable vocational choices (see Villwock et al., 1976; Holland, 1996). More precisely, when confronted with an appropriate strengths–behavior, one can easily tell which of the six core virtues is especially highly expressed and which is expressed to a lesser degree. For example, the enactment of creativity should be rated as a clear expression of wisdom.

Finally, there should be a higher consistency among the rating patterns in the core virtues; ratings of more appropriate acts should be more consistent than ratings of less appropriate acts.

In summary, we hypothesized:

H1a: Enactments based on the individual highest strength will be rated higher in the degree of strengths expression, the six core virtues (i.e., wisdom and knowledge, courage, humanity, justice, temperance, and transcendence), fulfillment, and moral and intellectual quality, and rated lower in immorality than descriptions based on lowest strengths.

H1b: Enactments based on excellently enacted strengths will be rated higher in the degree of strengths expression, the six core virtues (i.e., wisdom and knowledge, courage, humanity, justice, temperance, and transcendence), fulfillment, and moral and intellectual quality, and rated lower in immorality than descriptions based on typically enacted strengths.

H2a: Enactments based on the individual highest strength will show a higher differentiation in their core virtue ratings than descriptions based on lowest strengths.

H2b: Enactments based on excellently enacted strengths will show a higher differentiation in their core virtue ratings than descriptions based on typically enacted strengths.

H3a: Enactments based on the individual highest strength will show a higher agreement among raters who judge strengths-behaviors based on the same character strength than descriptions based on lowest strengths.

H3b: Enactments based on excellently enacted strengths will show a higher agreement among raters who judge strengths-behaviors based on the same character strength than descriptions based on typically enacted strengths.

### Method

#### Participants

A total of *N* = 230 German-speaking participants (81.3% women, 18.3% men, 0.4% other/not specified) aged 16 to 76 (*M* = 34.55 years; *SD* = 15.70) completed the study. This sample is comprised of 44.3% Germans, 40.4% Swiss, 10% Austrians, and 5.2% citizens from other countries. Most participants held a degree from a university or a university of applied sciences (39.1%) or held a diploma that would allow them to attend such universities (47.8%). In addition, 9.6% completed vocational training, 2.6% had completed secondary education, and 0.9% did not graduate from school.

#### Instruments

The *Character Behavior Task* served to collect strengths–behaviors. Participants were asked to recall situations in which they enacted their highest and lowest strengths in an excellent and typical way. First, participants’ highest and lowest character strengths were determined by the VIA-IS (Ruch et al., 2010). The VIA Inventory of Strengths (VIA-IS) assessed the 24 character strengths included in the VIA classification with 240 items (10 for each strength) on a five-point scale from 1 = very much unlike me to 5 = very much like me (for reliability and validity see Ruch et al., 2010). Then, participants were provided with definitions of the highest and lowest character strengths identified [taken from Ruch and Proyer (2015); based on Peterson and Seligman (2004) descriptions] and were asked to list five situations for each of the four conditions (i.e., highest/lowest character strength in an excellent/typical way). Participants were not informed that the selected character strengths represent their highest and lowest strengths. After that, they were asked to describe two of these situations (enactments) in more detail. They answered the following questions: Where did the situation take place? Who was there? What caused the situation, what was going on, which thoughts, feelings, and motivations did you have? How did the situation end? How can someone recognize that you used the strength? What relevant behaviors have been shown to exert the character strength? Participants wrote about two enactments per condition, which sums up to eight enactments in sum. Table 1 shows an example of each of the four conditions.

**TABLE 1:** An example of a description of enactments for the strength of creativity in all four conditions.

**Highest Strength excellently enacted**	**Lowest Strength excellently enacted**
I’ve invented new Zentangle patterns. I can’t paint, I spontaneously tried these patterns with a pen and had fun. I thought there had to be many more patterns—and found the art form centangle on the Internet. I painted patterns every day but didn’t show them to anyone. After half a year, I posted pictures on Facebook—and received positive feedback from all over the world. By mistake I invented my first own pattern. I had neither the intention to invent something new with this pattern nor with the following patterns. Every new pattern fills me with great joy and gratitude. These feelings multiply very much through the loving and appreciative comments on Facebook—and I almost burst with joy when other people paint my patterns! I paint patterns unintentionally. I can tell that I have used the strength by the fact that other people ask me for instructions for my pattern—so it’s something new that they can’t paint without further ado, but would like to. The behavior when using the strength is accompanied by unintentionality, joy of playing, fun in painting, innocence, and individuality; I follow my feelings.	I didn’t feel comfortable in the office and wanted a change. I was disturbed by the furnishing of the office, the positioning of my workstation, the many people walking around and the noise of the coffee machine. So, I suggested that my colleague move the office. We came up with a short plan and spontaneously rearranged the whole office. I feel much better now, and the problems and disruptive factors have been eliminated. I was very unbalanced earlier, couldn’t concentrate well and was often annoyed by the staff who didn’t care about us. The situation turned out to be that I feel very comfortable and my teammates and boss are also very satisfied. The office looks bigger and more open. I appreciate myself so much that I am not very creative, original and have great ideas. In everyday life, I may have great ideas such as cooking recipes, gift ideas, excursion ideas. Otherwise, I’m not very innovative. In this situation I used my ingenuity, because it was necessary (to solve the problem). My behavior was very deliberate. I compared different institutions and decided on the best idea.

**Highest Strength typically enacted**	**Lowest Strength typically enacted**

Before I got into software, I played with electronics. I built myself a digital clock, alone, in my nursery. Unfortunately, I had underestimated the quite high power requirements of the whole LEDs of the segment displays. If it was not 11:11 am, the clock’s power would not be enough. The solution was just awesome. I always have only a 7-segment display, so only one digit of the time displayed simultaneously. And so I switched so quickly between the segments that the human eye did not notice. It always looked as if all 4 digits were always lit. I had no notable thoughts and feelings. The problem was solved, it felt good.	This situation often happens in the evening when I come home hungry. Usually I cook for myself alone, because my roommate often works in the evening. I then inspect the fridge and see which food is still there. Then I think about which ingredients and which way of preparation I could use to cook a tasty dish. Often, I cook the best dishes under such circumstances. One recognizes the strength in which I managed to cook a tasty dish from ingredients or leftovers that do not seem to fit together without a ready-made recipe.

*Examples have been translated from German and abbreviated.*

In the *Virtue Judgment Instrument*, participants were asked to rate the degree of the six core virtues of wisdom, courage, humanity, justice, temperance, and transcendence in the strengths-behaviors described previously. They received definitions of the virtues based on Ruch and Proyer (2015) and rated the strengths–behaviors on a visual analog scale ranging from 0 (= the virtue is not shown at all) to 100 (= the virtue is shown to an extremely high extent).

Additionally, *ratings of the degree of strength expression* were collected. Participants were asked to rate the degree a strength was displayed in a particular strengths–behavior using a visual analog scale ranging from 0 (= the strength is not shown at all) to 100 (= the strength is shown to an extremely high extent).

In “*Ratings of Fulfillment, Intellectual and Moral Quality, and Immorality*”, participants were asked to rate their behaviors on a nine-point scale from 1 (“fulfillment/intellectual quality/moral quality/immorality not at all pronounced”) to 9 (“fulfillment/intellectual quality/moral quality/immorality extremely pronounced”). For ratings of intellectual and moral quality, participants were provided with descriptions of the concepts based on Nicomachean Ethics (Aristotle, 2000). The description of immorality was based on the study by Beermann and Ruch (2009). The instructions can be found in the online Supplementary Appendix B.

### Procedure

No ethics approval was required for this study according to the university guidelines. Participants were recruited via university mailing lists, psychology magazine websites, social platforms, and personal inquiry. Participants gave their written consent for participation and received partial course credit and/or an individual character strengths profile. Parts of the data (i.e., the virtue ratings with regard to the enactments of the highest character strengths) were reported previously in the study by Ruch et al. (2019). Participants first completed the VIA-IS, then they described eight situations in the *Character Behavior Task*, and finally they rated the strengths–behaviors described previously as explained in the *Virtue Judgment Instrument* and the *Ratings of Fulfillment, Intellectual and Moral Quality, and Immorality*. The order of strengths–behaviors to be rated was randomized.

### Results

Preliminary analyses of the intercorrelations of the dependent variables suggested positive relationships among most variables without indicating redundancy (Table 2).

**TABLE 2:** Intercorrelations of strengths expression, fulfillment, intellectual and moral quality, immorality, and the six core virtues in studies 1 and 2.

	**Strengths Expression**	**Fulfillment**	**Intellectual Quality**	**Moral Quality**	**Immorality**	**Wisdom**	**Courage**	**Humanity**	**Justice**	**Temperance**	**Transcendence**
Fulfillment	0.37***										
Intellectual Quality	0.26***	0.25***									
Moral Quality	0.15***	0.13***	0.29***								
Immorality	−0.12***	−0.15***	0.00	−0.07**							
Wisdom	0.26***	0.16***	0.50***	0.09***	−0.09***		0.12***	0.08***	0.21***	0.11***	0.01
Courage	0.22***	0.15***	0.14***	0.04	0.01	0.13***		0.08***	0.15***	0.07***	–0.02
Humanity	0.08**	0.04	0.03	0.44***	−0.12***	0.03	0.04		0.35***	–0.01	0.03
Justice	0.06*	–0.02	0.13***	0.37***	0.03	0.08**	0.09***	0.37***		0.13***	0.03
Temperance	0.07**	−0.10***	0.10***	0.17***	0.03	0.02	0.06*	0.05	0.16***		0.06**
Transcendence	0.13***	0.18***	0.08**	0.12***	−0.08**	0.12***	0.04	0.14***	0.04	0.01	

Below diagonal: Study 1, *N* = 203–230 (1588–1740 ratings). Above diagonal, Study 2: *N* = 113 (2712 ratings). Given are within-person correlations (Bland and Altman, 1995). Strength expression, fulfillment, intellectual and moral quality, and immorality were not analyzed in Study 2. *p < 0.05, **p < 0.01, ***p < 0.001.

The exception was immorality, which was negatively related to most variables. The degree of strengths expression went along with fulfillment, intellectual and moral quality, and all core virtue ratings. Fulfillment was positively related to intellectual and moral quality, wisdom, courage, and transcendence, and showed a small negative relationship to temperance. Intellectual quality showed positive relationships with moral quality, and all core virtue ratings, except for humanity. Moral quality was positively related to all core virtues, except for courage. Finally, most ratings of core virtues showed small positive correlations with each other, while the relationship between humanity and justice was of moderate size.

For the main analyses, we examined whether the levels of the virtue ratings, the differentiation among the ratings, and the agreement among participants were related to the rank of character strengths and the type of enactment. First, we analyzed whether the levels of the dependent variables (i.e., the degree of strengths expression, the six core virtues, fulfillment, intellectual quality, moral quality, and immorality) were related to the type of enactment and rank of character strengths. The sample sizes, means, and standard deviations of the dependent variables for the highest and lowest strengths in excellent and typical enactments are given in Table 3.

**TABLE 3:** Mean ratings of strengths expression, six core virtues, fulfillment, intellectual and moral quality, and immorality for the highest and lowest strengths excellently and typically enacted.

	**Highest strength**				**Lowest strength**			
	**Excellently enacted**		**Typically enacted**		**Excellently enacted**		**Typically enacted**	
	***M***	***SD***	***M***	***SD***	***M***	***SD***	***M***	***SD***
Strengths Expression	80.45	16.65	73.06	20.07	73.09	22.18	65.41	22.68
Wisdom	61.22	27.22	59.18	26.67	57.76	27.47	53.89	26.60
Courage	53.39	32.18	44.02	31.11	52.04	31.23	38.28	31.10
Humanity	58.06	31.90	55.55	33.17	57.17	32.62	52.36	31.98
Justice	40.64	31.21	38.02	32.66	39.52	33.01	40.01	33.51
Temperance	41.12	30.30	39.87	29.57	41.43	31.64	43.03	30.99
Transcendence	33.88	36.46	31.59	33.92	30.84	32.03	29.06	31.75
Fulfillment	6.48	1.98	6.25	1.68	5.99	1.97	5.53	1.89
Intellectual Quality	6.13	1.54	6.01	1.48	5.93	1.65	5.55	1.64
Moral Quality	5.96	1.77	5.63	1.79	5.77	1.94	5.61	1.89
Immorality	2.52	1.63	2.59	1.61	2.73	1.69	2.73	1.70

**N* = 203–230. Strengths expression, wisdom, courage, humanity, justice, temperance, and transcendence were rated on a scale ranging from 1 to 100, while fulfillment, intellectual quality, moral quality, and immorality were rated on a scale ranging from 1 to 9.*

Table 3 shows that regardless of the condition, the strengths–behaviors were considered fulfilling, of intellectual and moral quality (all mean ratings >5), and of low immorality (all mean ratings <3). Overall, a pattern can be observed: Mean values of the dependent variables decreased (and increased for immorality) from the highest strengths excellently enacted to the lowest strengths typically enacted. For courage, humanity, and moral quality, however, the mean values of the highest strengths and lowest strengths excellently enacted were followed by the highest strengths and lowest strengths typically enacted. Results for justice and temperance on the other hand showed a mixed pattern.

In order to test for differences among the conditions, we computed a set of factorial repeated measurement ANOVAs, with the type of enactment (typical vs. excellent) and the rank of character strengths (lowest vs. highest strength) as repeated factors, predicting the dependent variables (see Table 4).

**TABLE 4:** Results of factorial repeated measures ANOVA of relationships of character strengths rank (highest vs. lowest character strength) and enactment type (excellent vs. typical enactment) on the ratings of the strengths expression, six core virtues, fulfillment, intellectual and moral quality, and immorality.

	***F***	***p***	**Partial η ^2^**
Strengths Expression	*F*(1, 185)		
Rank	28.12	<0.001	0.132
Enactment	66.82	<0.001	0.265
Rank × Enactment	1.71	0.192	0.009
Wisdom	*F*(1, 188)		
Rank	10.394	0.001	0.052
Enactment	11.03	0.001	0.055
Rank × Enactment	0.86	0.354	0.005
Courage	*F*(1, 188)		
Rank	5.16	0.024	0.027
Enactment	59.74	<0.001	0.241
Rank × Enactment	1.24	0.267	0.007
Humanity	*F*(1, 188)		
Rank	0.69	0.408	0.004
Enactment	12.19	0.001	0.061
Rank × Enactment	1.49	0.224	0.008
Justice	*F*(1, 188)		
Rank	0.11	0.744	0.001
Enactment	2.27	0.133	0.012
Rank × Enactment	0.94	0.335	0.005
Temperance	*F*(1, 188)		
Rank	1.59	0.209	0.008
Enactment	0.19	0.663	0.001
Rank × Enactment	0.75	0.388	0.004
Transcendence	*F*(1, 188)		
Rank	3.79	0.053	0.020
Enactment	12.73	0.000	0.063
Rank × Enactment	0.06	0.804	0.000
Fulfillment	*F*(1, 208)		
Rank	17.21	<0.001	0.076
Enactment	16.49	<0.001	0.073
Rank × Enactment	2.54	0.113	0.012
Intellectual Quality	*F*(1, 204)		
Rank	13.39	<0.001	0.062
Enactment	12.20	0.001	0.056
Rank × Enactment	4.09	0.045	0.020
Moral Quality	*F*(1, 204)		
Rank	0.81	0.370	0.004
Enactment	11.86	0.001	0.055
Rank × Enactment	0.63	0.429	0.003
Immorality	*F*(1, 204)		
Rank	3.12	0.079	0.015
Enactment	0.69	0.408	0.003
Rank × Enactment	0.05	0.832	0.000

**N* = 189–208. Rank = Whether the described enactment was based on the individual lowest (=0) or highest (=1) ranking character strength. Enactment = Whether the described enactment represented a typical (=0) or an excellent (=1) display of the character strength.*

**FIGURE 1:**
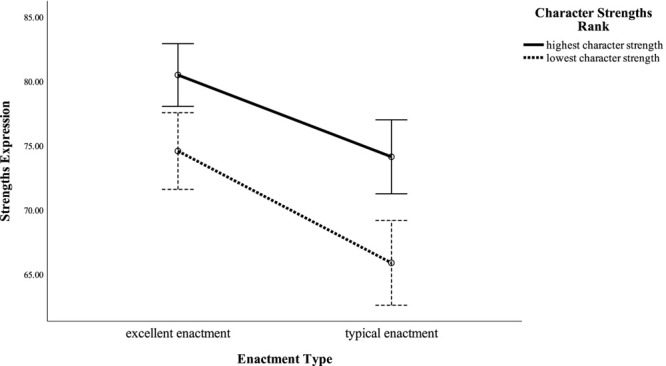
Degree of strengths expression for highest and lowest strengths under typical and excellent enactment conditions. Error bars represent 95% confidence intervals.

Table 4 shows that while there were no interactions between type of enactment and rank of character strengths in all dependent variables, both main effects were significant in two of the six core virtues, strengths expression, fulfillment, and intellectual quality: Participants indicated higher degrees of wisdom, courage, strengths expression, fulfillment, and intellectual quality for the situations in which they displayed their highest strengths compared to the situations in which they displayed their lowest strengths. For the same variables, ratings were higher when participants rated an excellent display of a strength than when they rated a typical display of a strength. Furthermore, in three dependent variables, only the main effect of enactment was significant: For humanity, transcendence and moral quality ratings of an excellent display of a strength were rated higher than a typical display of a strength. Figure 1 shows an example illustration of the results for the dependent variable strength expression.

Second, we analyzed whether the differentiation in the ratings of core virtues (i.e., the difference between the highest and lowest rating within a person) was related to the rank of character strengths and type of enactment. We computed a factorial repeated measurement ANOVAs with type of enactment (excellent vs. typical) and rank of character strengths (highest vs. lowest) as repeated factors, and the difference between the highest and the lowest rating of the core virtues as dependent variable. Results showed no interaction effect [*F*(1, 188) = 1.20, *p* = 0.274, η_*p*_^2^ = 0.006], but main effects for both enactment type [*F*(1, 188) = 7.07, *p* = 0.009, η_*p*_^2^ = 0.036] and rank of character strength [*F*(1, 188) = 17.52, *p* < 0.001, η_*p*_^2^ = 0.085], with a higher differentiation in ratings of excellent enactments and highest strengths. Thus, people who possessed a character strength to a high degree and described an excellent (as opposed to a typical) enactment showed a higher differentiation in the core virtues.

Third, for every character strength, we computed the interrater reliabilities (ICC1; one-way ANOVA random effects model, average measures) among the participants in their ratings of the enactments with regard to the six core virtues. Thereby, we obtained a score of agreement among participants who rated, for example, an excellent enactment for the highest strength of creativity. Overall, results suggested that agreement increased, when highest (vs. lowest) strengths were rated and when excellent (vs. typical) enactments were rated. The median of the ICCs across all 24 strengths were ICC = 0.70 (highest strength, excellent enactment), ICC = 0.65 (highest strength, typical enactment), ICC = 0.54 (lowest strength, excellent enactment), and ICC = 0.43 (lowest strength, typical enactment). Thus, as expected, there was the highest agreement with regard to the core virtues when people possessed a character strength to a high degree and described an excellent enactment.

### Discussion

The results of Study 1 partly confirmed our expectations and thereby validated the basic assumptions of “the layperson’s excellent enactment of highest strengths paradigm”: strengths–behaviors were rated higher in strengths expression, fulfillment, wisdom, courage, and intellectual quality, when the highest strengths and excellent enactments were considered. Exceptions were humanity, transcendence, and moral quality, which only related to the type of enactment, while justice, temperance, and immorality were unrelated to both the rank of the strengths and the type of enactment. Further, the results suggested that clearer distinctions in the ratings of the core virtues were made, while also a higher interrater reliability was obtained for excellent strengths–behaviors of highest-ranking strengths. Overall, we conclude that strengths–behaviors in which a high-ranking character strength was displayed in an excellent manner serve as a better basis for ratings of core virtues and for an examination of the VIA classification than behaviors based on general strengths–behaviors. So far, this is the first study that investigated which strengths enactments are best suited for the study of the assignment of character strengths to core virtues. We suggest that studies based on such a preselection of strengths–behaviors should provide more valid results than earlier studies that did not take this into account.

Therefore, we conducted a second study based on the results of Study 1 for examining the association of character strengths with core virtues. In Study 2, we further refined these excellent strengths–behaviors of highest-ranking strengths and asked a second group of participants (blind to the source of the descriptions) to rate these behaviors with regard to the six core virtues.

## Study 2

Study 2 intends to expand upon Ruch et al. (2019) study in which self-described strengths–behaviors were self-rated according to virtuousness, by using ratings of other people, unaffiliated with the people who provided the strengths–behaviors. We further refined the strengths–behaviors described in study 1, selecting only the most appropriate behaviors. We investigated whether an unrelated group of people rate the strengths–behaviors as virtuous in terms of the core virtues and whether these ratings are in line with the VIA classification—with the exception of some strengths where deviations from the VIA classification have been reported earlier. We hypothesized:

H1: The ratings of other people, unaffiliated with the people who provided the strengths–behaviors will recognize the core virtues in these descriptions (cutoff ≤ 40).

H2a: All enactments of character strengths (with the exception of forgiveness, gratitude, humor, teamwork, and leadership) will show a higher rating for the core virtue theoretically suggested in the VIA classification than for the other five core virtues averaged.

H2b: The enactments of the character strengths forgiveness, gratitude, humor, and teamwork will show higher ratings for the core virtue of humanity, and enactments of leadership will show higher ratings to the core virtue of courage, than for the other virtues averaged. These expected reclassifications are in line with earlier findings (Ruch and Proyer, 2015; Ruch et al., 2019).

### Method

#### Participants

The sample consisted of *N* = 113 German-speaking participants (76.1% women) with a mean age of 25.73 years (*SD* = 11.27, ranging from 18 to 81 years). The majority of participants (77.9%) were Swiss citizens, 16.8% were German citizens, and 5.3% had citizenship from different nations. The sample was rather well educated: 18.6% held a degree from a university or a university of applied sciences and 76.1% held a diploma allowing them to attend a university or a university of applied sciences, 4.4% completed vocational training, and 0.9% had completed primary or secondary school. Most of the participants did not know the VIA classification (79.6%), 12.4% have heard about the VIA classification, but did not know about the assignment of the character strengths to the virtues, and 4.4% did know the VIA classification and would be able to assign the character strengths to the virtues, if they were shown a list of the character strengths^1^.

#### Instruments

As in study 1, the *Virtue Judgment Instrument* (Ruch et al., 2019) was used. Participants rated the degree of the six core virtues of wisdom, courage, humanity, justice, temperance, and transcendence in the strengths–behaviors on a visual analog scale ranging from 0 (= the virtue is not shown at all) to 100 (= the virtue is shown to an extremely high extent).

##### Excellent signature strengths enactments rating task

Based on the strengths–behaviors presented in study 1 (total of 976 strengths–behaviors), the most appropriate strengths–behaviors were selected, which is in total 144 situations; i.e., six strengths–behaviors for each of the 24 character strengths. We selected the 144 strengths–behaviors from the 976 strengths–behaviors applying the following criteria: (1) The character strengths displayed (according to the person who provided the enactments) was recognized by at least one of two independent raters (in 65.27% of the behaviors, both raters recognized the strength). (2) The strengths–behaviors were rated unambiguous with regard to character strengths according to the two raters [i.e., as few character strengths as possible were recognized; in the selected strengths–behaviors, one (86.11%), two (9.03%), or three (4.86%) character strengths were recognized]. (3) The extent of strength expression was as high as possible (*M* = 86.58, *SD* = 14.55 across the selected enactments).

The resulting 144 strengths–behaviors were rated by the participants; each participant rated 24 enactments—one enactment for each character strength—with the *Virtue Judgment Instrument*. The order of strengths–behaviors to be rated was randomized.

#### Procedure

No ethics approval was required for this study according to the university guidelines. The study was conducted online, and participants were recruited via university mailing lists, psychology magazine websites, social platforms, and personal inquiry. Participants gave their written consent for participation. The participants were not compensated, but could receive partial course credit and/or a summary of the study results.

### Results

First, we analyzed the extent to which participants agreed in their evaluations of situations depicting character strengths with regard to the six core virtues, intellectual and moral quality, and immorality, by computing interrater reliabilities (ICC1; one-way ANOVA random effects model, average measures). Results suggested that agreement between participants is very high, ICC = 0.99 (across all variables). For a more detailed overview of the interrater reliabilities of all variables, see online Supplementary Appendix C.

Sample sizes, means, and standard deviations of the virtue ratings for all behavior descriptions are given in Table 5.

**TABLE 5:** Mean virtue ratings of the 24 character strengths in study 2.

	**Wisdom**		**Courage**		**Humanity**		**Justice**		**Temperance**		**Transcendence**	
	***M***	***SD***	***M***	***SD***	***M***	***SD***	***M***	***SD***	***M***	***SD***	***M***	***SD***
Creativity	**44.18**	**34.87**	27.77	30.22	27.35	32.85	11.81	22.45	11.60	22.45	16.81	26.04
Curiosity	**44.98**	**31.64**	**40.05**	**32.34**	32.05	32.50	12.35	23.41	24.53	29.06	18.89	27.77
Judgment	**51.67**	**28.40**	39.04	34.04	**40.39**	**33.01**	39.27	34.98	**43.46**	**35.37**	17.12	26.62
Love of learning	**64.47**	**28.53**	37.72	33.93	25.19	33.21	18.96	29.04	21.42	29.58	21.56	31.49
Perspective	**60.21**	**30.60**	29.03	30.76	**60.41**	**28.78**	36.39	34.38	20.74	27.89	15.42	25.80
Bravery	31.09	30.76	**69.00**	**30.77**	**40.73**	**38.10**	25.10	32.68	29.52	32.65	19.48	29.43
Perseverance	**51.35**	**29.50**	**48.04**	**36.89**	15.81	25.83	13.25	25.52	**44.68**	**34.74**	18.29	29.50
Honesty	37.97	32.17	**55.81**	**31.50**	**40.57**	**34.98**	**45.53**	**34.39**	28.53	34.15	20.04	29.95
Zest	36.83	30.35	**49.60**	**34.53**	36.66	32.88	17.01	30.00	24.89	33.18	26.21	33.12
Love	33.22	34.36	24.88	31.35	**66.55**	**31.45**	20.52	31.09	18.42	27.92	17.60	26.56
Kindness	**41.56**	**33.67**	34.73	33.05	**82.17**	**25.25**	**51.27**	**38.66**	29.75	34.05	31.62	35.84
Social intelligence	**51.74**	**31.06**	37.12	34.44	**75.24**	**26.23**	32.96	35.52	29.32	31.77	17.72	27.43
Teamwork	38.04	30.16	28.70	28.69	**53.63**	**34.46**	35.24	35.15	34.95	34.72	17.85	28.86
Fairness	**46.47**	**32.71**	35.94	33.77	**49.65**	**31.99**	**58.79**	**33.49**	31.69	35.03	18.20	28.60
Leadership	**63.33**	**26.73**	**42.33**	**32.40**	**49.31**	**32.85**	**42.19**	**35.25**	33.55	34.91	20.82	31.93
Forgiveness	**47.30**	**30.36**	**41.42**	**33.81**	**59.99**	**29.03**	**42.03**	**36.10**	37.29	31.82	20.06	28.17
Humility	28.50	29.35	11.50	19.78	**43.63**	**34.58**	27.69	31.79	39.58	38.04	16.58	27.43
Prudence	**50.97**	**33.09**	28.93	33.55	21.89	27.77	13.89	25.10	34.47	36.09	12.11	22.51
Self-regulation	39.61	32.25	29.41	33.59	15.46	26.06	7.84	18.99	**73.38**	**31.74**	14.14	26.17
Beauty	30.05	31.10	14.64	23.80	23.72	31.60	8.28	17.21	20.93	29.30	**43.94**	**35.86**
Gratitude	**48.76**	**30.88**	31.19	31.42	**50.42**	**35.52**	26.47	32.39	27.61	32.01	**43.39**	**37.42**
Hope	**40.96**	**32.78**	37.99	36.93	25.96	30.04	14.08	26.47	36.62	35.27	32.20	37.22
Humor	32.65	29.90	**40.82**	**32.30**	**54.63**	**31.39**	16.95	28.21	16.84	27.18	13.65	22.80
Spirituality	34.51	32.51	28.68	33.89	**44.97**	**34.45**	17.12	27.70	25.53	31.34	**72.27**	**33.43**

**N* = 113. The *N* refers to the number of raters per character strength, and not per enactment (for a detailed overview of the ratings, see Supplementary Appendix D). All means (and associated standard deviations) exceeding the cutoff (≥40) are printed in boldface. *Beauty* = Appreciation of Beauty and Excellence.*

For facilitating the interpretation, we used a score of ≥40^2^ as cutoff for being a good marker of a virtue. All strengths fulfilled the cutoff of at least one virtue, while several strengths exceeded the cutoff for two virtues (i.e., curiosity, perspective, bravery, social intelligence, humor, and spirituality), three virtues (i.e., judgment, perseverance, honesty, kindness, fairness, and gratitude), or four virtues (i.e., leadership and forgiveness). A total of 18 strengths were markers for the virtue they were originally assigned to by Peterson and Seligman (2004). The exceptions were teamwork, forgiveness, humility, prudence, hope, and humor.

We computed *t* tests for dependent samples to compare the mean ratings of the theoretically assigned virtue of a character strength with the mean ratings across the other five virtues (see Table 6).

**TABLE 6:** Comparison of ratings in the virtue that was suggested by Peterson and Seligman (2004) with the averaged ratings in the other virtues in study 2.

	***t* (df = *N* – 1)**	***p***	**Cohen’s *d*_*z*_**
Creativity	6.82	<0.001	0.91
Curiosity	6.42	<0.001	0.74
Open-mindedness	6.89	<0.001	0.64
Love of learning	12.91	<0.001	1.51
Perspective	11.02	<0.001	1.07
Bravery	12.35	<0.001	1.49
Persistence	5.67	<0.001	0.68
Honesty	7.76	<0.001	0.76
Zest	6.83	<0.001	0.74
Love	13.84	<0.001	1.60
Kindness	14.99	<0.001	1.75
Social intelligence	14.85	<0.001	1.70
Teamwork	0.22	0.825	0.02
Fairness	7.23	<0.001	0.81
Leadership	0.12	0.903	0.01
Forgiveness	–1.61	0.111	–0.19
Modesty	4.01	<0.001	0.47
Prudence	2.59	0.011	0.32
Self-regulation	16.30	<0.001	2.01
Beauty	6.64	<0.001	0.86
Gratitude	1.77	0.079	0.21
Hope	0.29	0.771	0.04
Humor	–8.86	<0.001	–0.88
Spirituality	11.31	<0.001	1.48

**N* = 113. *Beauty* = Appreciation of Beauty and Excellence.*

Table 6 shows that 17 of the 24 character strengths received higher ratings in the theoretically assigned virtue than in the mean of the other five virtues [*t*(112) ≥ 4.01, *p* < 0.001, Cohen’s *d*_*z*_ ≥ 0.47]. Humor, in contrast, received lower ratings in the theoretically assigned virtue than in the mean of the other five virtues, *t*(112) = -8.86, *p* < 0.001, Cohen’s *d*_*z*_ = -0.09.

Additionally, we compared our ratings with the assignment of character strengths to core virtues based on the empirical findings of Ruch and Proyer (2015) by reclassifying leadership to courage, and teamwork, forgiveness, gratitude, and humor to humanity. In this revised model, 21 out of the 24 character strengths marked the assigned virtue (i.e., ratings above the cutoff ≥ 40). The exceptions were humility, prudence, and hope.

When comparing the ratings of the assigned core virtue with the average of the other assigned virtues, results suggested a better fit for most of these strengths to this reclassified model. With the exception of leadership, these character strengths received higher ratings on the postulated virtue than on the averaged ratings of the other virtues [teamwork: *t*(112) = 7.51, *p* < 0.001, *d*_*z*_ = 0.79; forgiveness: *t*(112) = 7.85, *p* < 0.001, *d*_*z*_ = 0.91; gratitude: *t*(112) = 4.61, *p* < 0.001, *d*_*z*_ = 0.50; humor: *t*(112) = 10.79, *p* < 0.001, *d*_*z*_ = 1.17]. Thus, when taking earlier empirical findings into account, only leadership and hope did not fit to such a reclassified model.

Finally, we examined the overall convergence of the ratings with previous studies by correlating the matrix of the ratings (i.e., 24 character strengths × 6 virtues) in the present study with the VIA classification (coding the character strengths assigned to a virtue as 1 and the non-assigned strengths as 0) and the results of earlier studies. Results suggested a fair convergence with the VIA classification [*r*(142) = 0.51, *p* < 0.001] and the means reported in the second study of Ruch et al. (2019) [*r*(142) = 0.53, *p* < 0.001] and a good convergence with results by Ruch and Proyer (2015) [*r*(142) = 0.79, *p* < 0.001] and the first study by Ruch et al. (2019) [*r*(142) = 0.77, *p* < 0.001].

### Discussion

Study 2 applied “the layperson’s excellent enactment of highest strengths paradigm” and provided further validation of the VIA classification (Peterson and Seligman, 2004) by showing that the core virtues in strengths–behaviors can also be perceived by people who were not involved in the situation, where the character strength was displayed. While, as expected, lower ratings in the core virtues were obtained than when collecting ratings from the people who also provided the strengths–behaviors, the pattern of results mostly followed the expected pattern: The ratings of core virtues based on strengths–behaviors widely confirm the theoretical assignment of the VIA classification. When looking at absolute ratings, most (i.e., 18 out of the 24 character strengths) can be considered markers of the originally assigned virtue. At the same time, most character strengths (i.e., 15 out of the 24 character strengths) can be considered markers for more than one virtue, thus suggesting that a better fit of character strengths to core virtues would be obtained when taking a polytomous classification (i.e., allowing a strength to belong to more than one virtue), as discussed in-depth by Ruch and Proyer (2015) and Ruch et al. (2019). When looking at relative ratings (i.e., the ratings of a core virtue in relation to the average ratings of the other core virtues), 17 out of the 24 character strengths received higher ratings in the originally assigned virtue than for the other virtues.

For both absolute and relative ratings, a better fit was received when taking earlier empirical findings into account: When assigning character strengths to core virtues according to the findings of Ruch and Proyer (2015), 21 character strengths marked the corresponding virtue, while for 22 strengths, the assigned virtue was rated higher than the other virtues. Thus, only for the four strengths of hope (in both absolute and relative ratings), humility and prudence (absolute ratings), and leadership (relative ratings) was no fit to this revised classification found in the present study. It is hypothesized that this discrepancy is due to the fact that character strengths can correspond to multiple virtues (Ruch et al., 2019).

For hope, earlier studies (for a summary, see Ruch et al., 2019) suggested a good fit of hope to the virtue courage in addition to the originally assigned virtue of transcendence. In the present study, although the numerically highest ratings were obtained for the virtue of wisdom and knowledge, the ratings for the virtue of courage were rather close (i.e., the ratings of courage and wisdom differed by less than a tenth of a standard deviation in the ratings) and not far below the used cutoff of ≥ 40. The two strengths originally assigned to temperance, humility, and prudence received the numerically highest ratings in the present study for humanity, and wisdom and knowledge, respectively. Finally, also leadership (originally assigned to justice) was rated highest on wisdom. Again, all these relationships have already been reported in earlier studies (Ruch et al., 2019) in addition to the original assignments. Thus, while there are some discrepancies between the findings of the present study and earlier works, these are mostly small in size and widely confirm the previously reported patterns.

## General Discussion

The present studies extend knowledge about character strengths in two ways. First, the results suggested that when studying properties of character strengths based on strengths–behaviors, “the layperson’s excellent enactment of highest strengths paradigm” should be used. The paradigm states that for the investigation of character strengths, one should focus on behavior examples of people who possess a strength of interest to a high degree and displayed the strength in an excellent manner. With regard to “the layperson’s excellent enactment of highest strengths paradigm,” results showed that strengths–behaviors based on the highest character strength in an excellent way were rated higher in fulfillment, moral excellence (as suggested by two criteria of character strengths; contributing to fulfillments, and being morally valued in its own right), intellectual excellence, and the six core virtues than behavior examples based on typical enactments, or the lowest character strength. Furthermore, a higher differentiation in the ratings and a higher agreement among raters was found. Thus, we conclude that the “layperson’s excellent enactment of highest strengths paradigm” offers a valuable pathway for studying basic characteristics of character strengths.

Interestingly, also displays of the lowest strengths in typical enactments were, on average, rated as rather fulfilling (means were above the theoretical scale midpoint ranging from “fulfillment not at all pronounced” to “fulfillment extremely pronounced”). Thus, while it is more fulfilling to display a high-ranking strength in an excellent manner, displaying a low-ranking strength in a typical manner can also be considered somewhat fulfilling. This finding confirms Peterson and Seligman’s (2004) hypothesis about fulfillment: strengths indeed contribute to fulfillment; both high-ranking and low-ranking strengths do so. Furthermore, this result might partially explain why experimental studies that contrasted interventions based on the highest-ranking strengths with interventions based on the lowest-ranking strengths usually did not find any differences between the interventions with regard to their effectiveness for increasing well-being (e.g., Rust et al., 2009; Proyer et al., 2015; see also Ruch et al., in press).

The second main contribution of the present set of studies is that they provide further empirical information on the assignment of character strengths to virtues by applying “the layperson’s excellent enactment of highest strengths paradigm” and using different groups of people for providing the strengths–behavior’s and the core virtue ratings. Overall, the results converged well with the assignment suggested in the VIA classification for most of the 24 character strengths. However, the convergence increased when taking into account earlier empirical findings and re-assigning the strengths of teamwork, leadership, forgiveness, gratitude, and humor to other virtues than originally suggested by Peterson and Seligman (2004). Although some discrepancies between the expected and the data-driven assignment of strengths to virtues remained, similar findings have already been noted in earlier studies.

Overall, there is growing evidence that while the assignment of character strengths in the original classification seems to withstand empirical testing for most strengths, some adjustments should be considered. We do not suggest specific changes at the present moment but instead encourage further research using different methods and approaches before summarizing existing evidence and concluding on a revised, empirically backed classification. Nonetheless, we want to summarize the current state of findings. For the three character strengths of gratitude, forgiveness, and humor, the present study and all earlier empirical studies on this subject (Ruch and Proyer, 2015; Ruch et al., 2019) unequivocally suggest a reassignment to the core virtue of humanity. Thus, these three strengths seem to be the most dominant candidates for a future reassignment. The next best candidate would be teamwork, with most studies pointing to a better assignment to the core virtue of humanity. For leadership, the case is less clear: Across several studies, no dominant assignment to a virtue emerged; instead, leadership seems to fit well to several core virtues, predominantly wisdom and knowledge, courage, humanity, and justice. Recently revised versions of the self-report instruments for assessing character strengths (McGrath and Wallace, 2019) presumably even further reduced the associations of leadership to the core virtue of justice due to focusing more strongly on general leadership abilities than on fair leadership, as in the original instrument (Peterson and Seligman, 2004). Similarly, there are some inconsistencies with regard to the findings for prudence, humility, and hope. In earlier studies, prudence and humility yielded a good fit to the originally assigned virtues of temperance but also the virtues of wisdom and knowledge (prudence), and humanity (humility), which was also confirmed in the present study. For hope there is some evidence for its original classification (transcendence), but also to other virtues, including courage and wisdom, as in the present study.

Furthermore, one consequence of a potential reclassification of character strengths should be discussed: When using a dichotomous assignment, as in the original VIA classification (i.e., each character strength is assigned to only one core virtue), a potential reassignment of forgiveness and leadership would leave the core virtue of justice with only one character strength, namely, fairness. This would contradict the idea that the character strengths assigned to one virtue represent *different* routes for displaying this virtue, and only one such route would remain for the virtue of justice. Instead, due to their empirical co-occurrence (Ruch et al., 2019), and their strong conceptual similarity, as already noted by Peterson and Seligman (2004), one might consider unifying the virtues of humanity and justice to a general virtue related to improving other’s welfare. On the other hand, one might also argue that the differences between the two virtues are rather subtle and it is therefore difficult to disentangle the two virtues by the applied methods. Thus, further research with a special focus on the differences between humanity and justice, ideally using more elaborate descriptions of the core virtues, is encouraged. Furthermore, there is still a need to study the mechanism between character strengths and virtues. As Miller (2019) states, the characterization of the link between character strengths and virtues is very compact in Peterson and Seligman (2004) book, and because of this, various interpretations about the link between both are possible. Thus, further research on the relationships between the character strengths (e.g., humor) and the associated virtues (e.g., humanity) is warranted.

### Limitations and Directions for Future Research

Of course, the results presented here need to be interpreted in light of some limitations. First, the strengths–behaviors investigated are based on remembered experiences. This could have led to shortcomings in the recalling process. For example, it is possible that participants could have remembered the enactments described in a more positive way than they were, which in turn could have an influence on the ratings of all dependent variables. In future studies, other methods could be applied, such as experience sampling methods, journaling, or behavioral observations, to obtain a more accurate impression of the enactments and more accurate ratings of the dependent variables. Second, we analyzed enactments of character strengths and found that, in study 1, at least one virtue was recognized in 93.17% of the enactments of highest strengths and in 88.90% of the enactments of lowest strengths (cutoff: ratings ≥ 50). In study 2, strangers recognized at least one virtue in 90.97% of the enactments (cutoff: ratings ≥ 40). Thus, we conclude that strengths enactments are in most cases considered virtuous in terms of the six core virtues. However, in future studies, the comparison with enactments of other traits, motivations, interests, or performances is needed. While we do not expect that the enactments of other traits will be virtuous, fulfilling, intellectually and morally excellent, or reach the same level of virtuousness, fulfillment, and both intellectual and moral excellence, this hypothesis will need to be tested in a future study. Third, specific enactments of character strengths as used in the present study were not always “pure”; for several enactments (i.e., about 14% of the enactments in study 2), the raters perceived a second or a third character strength in the enactment. While we did not find evidence for a systematic bias in the results, it cannot be ruled out that this also affected our findings. Furthermore, while we found that enacting character strengths is perceived as intellectually and morally excellent, we did not examine whether character strengths are morally valued in their own right, without the absence of beneficial outcomes. Fourth, the characteristics of our samples could be seen as a further limitation of the study, particularly the fact that the participants are mainly highly educated women. Highly educated people, as well as women, might express their character strengths in a different way than less educated people or men. It could be hypothesized that well-educated people express different virtuous behavior than less educated people. Furthermore, higher educated people may have more facilities in recognizing virtuousness in the enactment of character strengths compared to less educated people, and it could be further debated whether or not women and men rate the degree of virtuousness differently from one another. In future studies, it would be profitable to study whether less educated people or men report qualitatively different situations of strengths enactment, and whether their virtue ratings differ.

Further research using “the layperson’s excellent enactment of highest strengths paradigm” is also needed. First, the present study was done with educated participants in one language region and is therefore not generalizable. We do not know how excellent enactment of a strength might vary with age, social class, or education. More importantly, culture might influence the results and one can easily imagine that individualistic cultures might develop different patterns than collectivistic one. Likewise, religion might play a role. More precisely, basic characteristics of character strengths such as the 12 criteria (e.g., fulfillment, morally valued) or characteristics of signature strengths are recommended to be studied by interviewing people who possess the character strength to a high degree and enact that strength in an excellent way, as these people can be seen as the natural experts on character strengths. Furthermore, we also encourage to apply the paradigm when developing character interventions or programs promoting virtues. People who do possess the character strength to a high degree and enact that strength in an excellent way will provide valid information on how strengths actually lead to virtuous behavior and how virtues can be promoted.

## Conclusion

In conclusion, the present set of two studies introduced and evaluated “the layperson’s excellent enactment of highest strengths paradigm.” This paradigm states that focusing on excellent behavior examples of people who possess a strength to a high degree yields more appropriate results with regard to basic properties of character strengths than when considering other behavior examples. Results widely confirmed this assumption and suggest that the paradigm offers a valuable approach for future research endeavors when studying fundamental questions with regard to character strengths. Further, the studies provided further empirical information on the assignment of character strengths to virtues based on a more rigorous approach than previous studies, and mostly supported previous findings on convergence and discrepancies with regard to the original VIA classification.

## Data Availability Statement

The datasets generated for this study are available on request to the corresponding author.

## Ethics Statement

This study was carried out in accordance with the recommendations of the Swiss Psychological Association with written informed consent from all subjects. All subjects gave written informed consent in accordance with the Declaration of Helsinki. According to the university guidelines (University of Zurich), the present study did not require a formal approval.

## Author Contributions

FGi, WR, and FGa conceptualized and designed the work, analyzed and interpreted the data, critically revised the manuscript, and approved the final version of the manuscript. FGi collected the data. FGi and FGa drafted the manuscript. All authors contributed to the article and approved the submitted version.

## Conflict of Interest

WR is a Senior Scientist for the VIA Institute on Character, which holds the copyright to the VIA Inventory of Strengths.

The remaining authors declare that the research was conducted in the absence of any commercial or financial relationships that could be construed as a potential conflict of interest.

## Footnotes

1 Analyzing the data without participants with a good knowledge of the VIA classification did not change the results; therefore, all analyses were conducted including these participants.

2 While an earlier study (Ruch et al., 2019) used a cutoff of ≥50 for being a good marker of a virtue, we lowered the cutoff because we obtained, as expected, overall lower ratings than when using self-reports.
