# Supplementary material for: Does the Excellent Enactment of Highest Strengths Reveal Virtues?
Source: Front Psychol. 2020 Jul 22;11:1545. doi: 10.3389/fpsyg.2020.01545 (PMC7387663; doi:10.3389/fpsyg.2020.01545)
Supplement: Supplementary file 1 [file Table_1.DOCX]

Online Supplementary Material

# Appendix A

*The VIA-Classification of six virtues and 24 character strengths*

| Virtue I. Wisdom and knowledge: cognitive strengths that entail the acquisition and use of knowledge. | |
| --- | --- |
| (1) | creativity: thinking of novel and productive ways to do things |
| (2) | curiosity: taking an interest in all of ongoing experience |
| (3) | open-mindedness: thinking things through and examining them from all sides |
| (4) | love of learning: mastering new skills, topics, and bodies of knowledge |
| (5) | perspective: being able to provide wise counsel to others |
| Virtue II. Courage: emotional strengths that involve the exercise of will to accomplish goals in the face of opposition, external or internal. | |
| (6) | bravery: not shrinking from threat, challenge, difficulty, or pain |
| (7) | persistence: finishing what one starts |
| (8) | honesty: speaking the truth and presenting oneself in a genu- ine way |
| (9) | zest: approaching life with excitement and energy |
| Virtue III. Humanity: interpersonal strengths that involve “tending and befriending” others. | |
| (10) | love: valuing close relations with others |
| (11) | kindness: doing favors and good deeds for others |
| (12) | social intelligence: being aware of the motives and feelings of self and others |
| Virtue IV. Justice: civic strengths that underlie healthy community life. | |
| (13) | teamwork: working well as member of a group or team |
| (14) | fairness: treating all people the same according to notions of fairness and justice |
| (15) | leadership: organizing group activities and seeing that they happen |
| Virtue V. Temperance: strengths that protect against excess. | |
| (16) | forgiveness: forgiving those who have done wrong |
| (17) | modesty: letting one’s accomplishments speak for themselves |
| (18) | prudence: being careful about one’s choices; not saying or doing things that might later be regretted |
| (19) | self-regulation: regulating what one feels and does |
| Virtue VI. Transcendence: strengths that forge connections to the larger universe and provide meaning. | |
| (20) | appreciation of beauty and excellence: noticing and appreciating beauty, excellence, and/or skilled performance in all domains of life |
| (21) | gratitude: being aware of and thankful for the good things that happen |
| (22) | hope: expecting the best and working to achieve it |
| (23) | humor: liking to laugh and joke; bringing smiles to other people |
| (24) | religiousness: having coherent beliefs about the higher pur- pose and meaning of life |

# Appendix B

*Definitions of The Concepts of Intellectual and Moral Quality, and Immorality*

| **Concept** | **Definition (German)** | **Definition (English)** |
| --- | --- | --- |
| Intellectual Quality | Intellektuelle Qualität bezieht sich auf das Denkvermögen und zeichnet sich durch ein hohes Ausmass an Wissen, Verstand und Lebenserfahrung aus. Intellektuelle Qualität hilft dabei, konkrete Situationen richtig zu beurteilen und geeignete Mittel und Wege zu finden, um das Richtige zu tun. | Intellectual quality refers to the ability to think, and is characterized by a high degree of knowledge, understanding and life experience. Intellectual quality helps to judge concrete situations correctly and to find suitable ways and means to do the right thing. |
| Moral Quality | Moralische Qualität zeichnet sich durch ein hohes Ausmass an Selbstlosigkeit, Nächstenliebe oder Selbstbeherrschung aus. Moralische Qualität hilft dabei, moralisch und ethisch richtig und verantwortungsvoll zu handeln und das Wohl Anderer im Sinn zu haben. | Moral quality is characterized by a high degree of selflessness, charity or self-control. Moral quality helps to act morally and ethically correct and responsible and to have the well-being of others in mind. |
| Immorality | Lasterhaftigkeit bzw. eine Untugend beschreibt etwas tadelnswertes, ein schädliches Handeln, eine schlechte Angewohnheit, von der jemand beherrscht wird; Verhaltensweisen, die zum Schaden des Einzelnen bzw. von Gruppen oder Gesellschaften führen bzw. führen sollen. Es geht dabei nicht um pathologische Verhaltensweisen, sondern die moralische bzw. ethische Wertung steht im Vordergrund. | Immorality describes something reprehensible, a harmful action, a bad habit which dominates someone, behavior which leads or is to lead to the detriment of the individual or of groups or societies. It is not a question of pathological behavior, but of moral or ethical evaluation. |

# Appendix C

*Intraclass-Coefficients and 95% - Confidence Intervals of virtues, intellectual and moral quality, and immorality*

|  | ICC (1, 113) | 95% - Confidence Intervals |
| --- | --- | --- |
| All | .986 | .980 – .987 |
| Wisdom | .942 | .863– .958 |
| Courage | .954 | .899 – .969 |
| Humanity | .979 | .955 – .986 |
| Justice | .971 | .935 – .980 |
| Temperance | .953 | .899 – .969 |
| Transcendence | .970 | .926 – .977 |
| Intellectual Quality | .935 | .859 – .957 |
| Moral Quality | .967 | .928 – .978 |
| Immorality | .877 | .686 – .904 |

*Note. N* = 113. All = Wisdom, Courage, Humanity, Justice, Temperance, Transcendence, Intellectual Quality, Moral Quality, and Immorality at the same time. ICC = ICC(1, 113), one-way random effects model, average of multiple raters.

# Appendix C

*Number of raters in each situation.*

|  | *N* in situation: | | | | | |
| --- | --- | --- | --- | --- | --- | --- |
|  | 1 | 2 | 3 | 4 | 5 | 6 |
| Creativity | 15 | 20 | 21 | 13 | 19 | 25 |
| Curiosity | 17 | 15 | 25 | 16 | 18 | 22 |
| Open-mindedness | 19 | 18 | 15 | 23 | 17 | 21 |
| Love of learning | 24 | 19 | 21 | 15 | 20 | 14 |
| Perspective | 14 | 23 | 18 | 18 | 23 | 17 |
| Bravery | 22 | 15 | 18 | 22 | 18 | 18 |
| Persistence | 20 | 17 | 17 | 20 | 21 | 18 |
| Honesty | 24 | 20 | 24 | 20 | 13 | 12 |
| Zest | 20 | 23 | 14 | 19 | 16 | 21 |
| Love | 19 | 26 | 20 | 19 | 11 | 18 |
| Kindness | 20 | 15 | 12 | 16 | 24 | 26 |
| Social intelligence | 15 | 21 | 16 | 20 | 16 | 25 |
| Teamwork | 13 | 14 | 16 | 24 | 22 | 24 |
| Fairness | 26 | 19 | 18 | 14 | 20 | 16 |
| Leadership | 20 | 25 | 18 | 21 | 13 | 16 |
| Forgiveness | 27 | 19 | 17 | 16 | 17 | 17 |
| Modesty | 20 | 22 | 12 | 25 | 19 | 15 |
| Prudence | 21 | 19 | 22 | 15 | 17 | 19 |
| Self-regulation | 27 | 18 | 12 | 16 | 19 | 21 |
| Beauty | 19 | 17 | 23 | 18 | 22 | 14 |
| Gratitude | 13 | 13 | 16 | 23 | 25 | 23 |
| Hope | 21 | 17 | 20 | 25 | 12 | 18 |
| Humor | 16 | 18 | 17 | 19 | 21 | 22 |
| Spirituality | 16 | 19 | 21 | 16 | 22 | 19 |

*N* = 113.
